# Scaffold compound L971 exhibits anti‐inflammatory activities through inhibition of JAK/STAT and NFκB signalling pathways

**DOI:** 10.1111/jcmm.16609

**Published:** 2021-05-20

**Authors:** Mengyuan Li, Yu Yan, Xinxin Zhang, Yidan Zhang, Xiaohan Xu, Lei Zhang, Liangliang Lu, Jie Wang, Yazhuo Zhang, Qiaoling Song, Chenyang Zhao

**Affiliations:** ^1^ School of Medicine and Pharmacy Ocean University of China Qingdao China; ^2^ Innovation Platform of Marine Drug Screening & Evaluation Qingdao National Laboratory for Marine Science and Technology Qingdao China; ^3^ School of Life Science Lanzhou University Lanzhou China

**Keywords:** anti‐inflammation, JAK/STAT, NFκB, sepsis shock, transcriptome sequencing

## Abstract

JAK/STAT and NFκB signalling pathways play essential roles in regulating inflammatory responses, which are important pathogenic factors of various serious immune‐related diseases, and function individually or synergistically. To find prodrugs that can treat inflammation, we performed a preliminary high‐throughput screening of 18 840 small molecular compounds and identified scaffold compound L971 which significantly inhibited JAK/STAT and NFκB driven luciferase activities. L971 could inhibit the constitutive and stimuli‐dependent activation of STAT1, STAT3 and IκBα and could significantly down‐regulate the proinflammatory gene expression in mouse peritoneal macrophages stimulated by LPS. Gene expression profiles upon L971 treatment were determined using high‐throughput RNA sequencing, and significant differentially up‐regulated and down‐regulated genes were identified by DESeq analysis. The bioinformatic studies confirmed the anti‐inflammatory effects of L971. Finally, L971 anti‐inflammatory character was further verified in LPS‐induced sepsis shock mouse model in vivo. Taken together, these data indicated that L971 could down‐regulate both JAK/STAT and NFκB signalling activities and has the potential to treat inflammatory diseases such as sepsis shock.

## 1 INTRODUCTION

Inflammation is an innate immune response against trauma, infection, tissue injury or noxious stimuli.^1^ The inﬂammatory process is managed by various immune cells such as neutrophils and macrophages and by vast majority of signalling pathways.^2^ Sepsis is a syndrome with multiple organ or tissue damage caused by systematic inflammatory reactions resulting from infection and trauma. Recent advances have started to reveal the highly complex pathophysiology of sepsis. Abnormalities in cytokine receptor signalling pathways are responsible for inflammatory sepsis.^3^ Numerous pro‐inflammatory cytokines, such as interleukin 1 (IL1), interleukin 6 (IL6) and tumour necrosis factor α (TNFα), are produced and utilize complex signalling cascades, especially Janus tyrosine kinase/ signal transducer and activator of transcription (JAK/STAT) and nuclear factor kappa B (NFκB) signalling to exert their biological effects in sepsis shock. When cells are activated with exogenous stimuli such as endotoxin, gram‐positive bacterial products (eg peptidoglycans and lipoteichoic acid), cytokines (eg TNFα and IL1) and other physical and chemical stressors, STATs and NFκB become activated in the cytoplasm and then translocate into the nucleus where they activate transcription of many inflammatory mediators.^4^ Besides, certain pathogen recognition receptors (PRRs) sensing stimuli rapidly activate NFκB, Interferon regulatory factor 3 and 7 (IRF3/7), leading to secretion of cytokines and chemokines including IL6 and interferons, which in turn activate JAK/STAT signalling. Moreover, some cytokine transcription is modulated by NFκB and STAT complex.^5^ Therefore, dual targeting JAK/STAT and NFκB might provide more efficient anti‐inflammatory effects.

As classical anti‐inflammatory therapy utilizing non‐steroidal anti‐inflammatory drugs and cytochrome c oxidase 1/2 (COX1/2) inhibitors exhibits a wide spectrum of potential risks, the advanced generation of novel anti‐inflammatory drugs primarily acting against pro‐inflammatory mediator production are being developed. Among these biological targets, JAK/STAT and NFκB signalling molecules represent the most promising pathways for achieving optimal therapeutic response with minimal side effects.^6^

Numerous compounds acting directly on the NFκB protein complexes or NFκB‐related signalling pathways were developed including plant‐derived agents (extracts and essence), steroid‐based compounds and several small‐molecule mediators jointly composing a large therapeutic group. Some of small‐molecular NFκB inhibitors are approved and launched into clinical practice to treat various inflammatory diseases such as inflammatory bowel disease, rheumatoid arthritis and non‐specific inflammation.^7^ There are also several small molecule inhibitors targeting JAK kinase and STAT function, which are already launched on the market or currently evaluated in different clinical trials against various inflammatory conditions.^8^, ^9^ As inhibitors targeting these two pathways both exhibited similar anti‐inflammatory applications, and these two pathways frequently crosstalk with each other in various conditions,^10^, ^11^, ^12^ synergetic drug combination or drugs with dual inhibition activity might be more efficient for the treatment of inflammatory disease such as sepsis shock. In a very recently reported study, combination of sitagliptin and tofacitinib significantly ameliorates adjuvant induced arthritis via modulating the interaction between JAK/STAT and NFκB signalling,^13^ verifying the feasibility of dual targeting drug or drug combination development.

In the current study, a dual STAT and NFκB‐based luciferase drug screening system was constructed to screen the compounds which meet this criterion. Total of 18 840 small molecular compounds were screened using this reporter system, and a scaffold compound named L971‐0101 (L971) was identified as lead compound targeting both JAK/STAT and NFκB signalling. It can potently inhibit constitutive and cytokine‐induced activation of STAT1, STAT3 and NFκB and their downstream gene expression in both cancer cells and macrophages. To comprehensively uncover L971 function, the high‐throughput RNA sequencing was employed in macrophages treated with LPS or LPS+L971. The analysis results clearly demonstrated that L971 significantly down‐regulates LPS‐induced JAK/STAT and NFκB signalling activities, immune cell activation and inflammatory diseases such as sepsis shock. As an anti‐inflammatory compound, L971 was proved to alleviate LPS‐induced sepsis shock mouse model in vivo, suggesting its therapeutic potential for sepsis treatment.

## 2 MATERIALS AND METHODS

### 2.1 Antibodies and reagents

Primary antibodies used in this study were detailed in Table S1. Cell lysis buffer (Cat. 9803) were from Cell Signaling Technology. Recombinant human or mouse IL6 (Cat. 216‐16; 200‐06), TNFα (Cat. 315‐01A; 300‐01A) and IFNβ (Cat. 300‐02BC) were obtained from PeproTech, whereas protease inhibitor (Cat. 11836145001) and phosphorylation inhibitor cocktail (Cat. 4906837001) tablets were from Roche Diagnostics. Horseradish peroxidase‐conjugated secondary antibodies (Cat. abs20001; abs20002) were from Absin. Kolliphor® HS 15 (HS‐15) (Cat. 42966), and LPS (Cat. 916374) were bought from Sigma. Mouse granulocyte colony‐stimulating factor (G‐CSF) ELISA Kit (Cat. ab197743) was bought from Abcam. The chemical libraries were purchased from TargetMol or The National Center for Drug Screening (China). The detailed compound information was listed in Table S2. The scaffold compound L971‐0101 (L971) was acquired from TargetMol.

### 2.2 Plasmid and reporter system construction

A sequence containing 16 × SIE (8 × 5′‐TTCCTGTAA‐3′ and 8 × 5′‐TTCCCGTAA‐3′) plus 1 × NFκB (5’‐GGGAATTTCC‐3’) binding elements with one TATA box was inserted into pGL4.20 between KpnI and HindIII. The SIE‐NFκB‐luc puromycin construct was transfected into A549 cell line. Forty‐eight hours after transfection, cells were selected with 5 mg/ml puromycin for 2 weeks, then 2.5 mg/ml for another 2 weeks. Clone SKA‐Ⅱ was picked up and analysed.

### 2.3 Cell lines and cell culture

HeLa, DU145, A549 and THP‐1 cells were obtained from the American Type Culture Collection (ATCC). HeLa, peritoneal macrophages and SKA‐Ⅱ cells were incubated in Dulbecco's modified Eagle's medium (DMEM), and DU145 and THP‐1 cells were cultured in Roswell Park Memorial Institute (RPMI) 1640 medium. These media were supplemented with 10% foetal bovine serum (FBS, Gibco), penicillin (100 IU/ml) and streptomycin (100 mg/ml), and the cells were maintained at 37°C in humidified incubators containing 5% CO_2_. All the cell lines were authenticated by STR profiling and tested without mycoplasma contamination.

### 2.4 Primary peritoneal macrophage isolation

Detailed protocol to harvest primary mouse peritoneal macrophage was reported previously.^14^ Briefly, 38.5 g of the BBL^™^ Thioglycollate Medium Brewer Modified powder (BD Biosciences, Cat. 211716) was dissolved in 1 L of purified water with frequent agitation, autoclaved at 121°C for 15 minutes and stored at 4°C for at least 3 months. Mice were injected intraperitoneally with 1 ml of aged thioglycolate. Seventy‐two hours later, mice were killed, and peritoneal macrophages were harvested by flushing peritoneal cavity.

### 2.5 Animals

Male C57BL/6 mice (SPF degree, 6 weeks old) were purchased from Beijing Vital River Laboratory Animal Technology Co., Ltd. They were maintained in the specific‐pathogen‐free laboratory animal room with a 12 hours light dark cycle. All animal procedures were approved by the Committee of Experimental Animals of School of Medicine and Pharmacy, Ocean University of China (OUCSMP‐20200701) and conformed to the Guide for the Care and Use of Laboratory Animals published by the United States National Institutes of Health (NIH Publication No 85‐23, revised 1996).

### 2.6 Luciferase reporter assay

SKA‐Ⅱ cells (1 × 10^4^/well) were seeded into white 96‐well plates (Corning) and incubated overnight at 37℃ in an incubator containing 5% CO_2_. These cells were then treated with the either vehicle or L971 at the indicated concentrations for 24 hours. Luciferase activity was determined using Promega luciferase kits (Cat. E2510) and detected by a SpectraMax^®^ L microplate reader (Molecular Devices).

### 2.7 Western blotting

Immune cells or tumour cells treated with 2.5, 5, 7.5 and 15 μM of L971 with or without cytokine treatment: 20 ng/ml IL6, 50 ng/ml IFNβ and 20 ng/ml TNFα for 10 minutes, or 100 ng/ml LPS for 0.5 hours. Cells were washed twice with cold PBS and harvested in lysis buffer containing protease and phosphatase inhibitors. Total of 20 μg protein lysates were resolved by SDS‐PAGE electrophoresis gel and transferred onto nitrocellulose membranes (GE Healthcare, Cat. 10600034). After blocking with nonfat milk solution (with 0.5‰ Tween‐20), the membranes were probed with primary antibodies at 4℃ overnight and then incubated with horseradish peroxidase‐conjugated secondary antibodies for 2 hours at room temperature. Immune complexes were detected with an Immobilon™ western chemiluminescence horseradish peroxidase substrate (Millipore, Cat. WBKLS0500) and photographed with a Tanon 5200 imaging system. The quantification was managed by ImageJ software.

### 2.8 Real‐time PCR measurement

Cells were treated the same as described in 2.7 Western blotting. Total RNA from cultured cells was extracted with RNAisoPlus (TaKaRa, Cat. 9109). Reverse transcription was performed with a PrimeScript™ RT reagent kit (Roche, Cat. RR037A) and genomic DNA Eraser, and cDNA samples were amplified using SYBR Green (Roche, Cat. 4913914001) in StepOne Plus Real‐Time PCR System (Applied Biosystems). The primer sequences for RT‐PCR were previously described.^15^, ^16^

### 2.9 Mouse sepsis model

Mice were randomly divided into different groups and intraperitoneally (i.p.) treated with vehicle (10% HS‐15 + 2% DMSO in PBS buffer) or L971 at 2.5 or 5 mg/kg. Twelve hours later, all the mice were challenged i.p. with 10 mg/kg LPS. Body temperature and mouse survival were monitored.

### 2.10 Transcriptome sequencing

Mouse peritoneal macrophages were isolated and treated with or without L971 (15 μM) for 0.5 hours, followed by vehicle or LPS (100 ng/ml) challenge for additional 4 hours. Total RNA was isolated using TRIzol (Thermo Fisher Scientific). After extracting the total RNA of the sample and digesting the DNA with DNase, the magnetic beads with Oligo (dT) were used to enrich eukaryotic mRNA. cDNA library construction and sequencing were performed using the Illumina Hiseq Xten platform. After passing the quality inspection, raw data (raw reads) were processed using Trimmomatic. The reads containing ploy‐N and the low‐quality reads were removed to obtain clean reads. Then, the clean reads were mapped to reference genome using Hisat2.^17^ The FPKM value of each gene was calculated using cufflinks,^18^ and the read counts of each gene were obtained by htseq‐count.^19^ Differentially expressed genes (DEGs) were identified using the DESeq. *P*‐value <.05 and fold change >2 or fold change <0.5 were set as the threshold for significantly differential expression. Hierarchical cluster analysis of DEGs was performed to explore genes expression pattern. KEGG pathway enrichment analysis,^20^ PPI^21^ and IPA^22^ analysis of DEGs were, respectively, performed.

### 2.11 Statistical analysis

All the histograms and line charts were made by GraphPad Prism 8.0.1. Results were graphed as mean ± SEM. Statistical significance was calculated by a two‐tailed Student's t test for comparisons. Significant differences are indicated as * *P*‐value <.05.

## 3 RESULTS

### 3.1 **L971 was identified as an inhibitor of JAK/STAT and NF**κ**B signalling**

**FIGURE 1:**
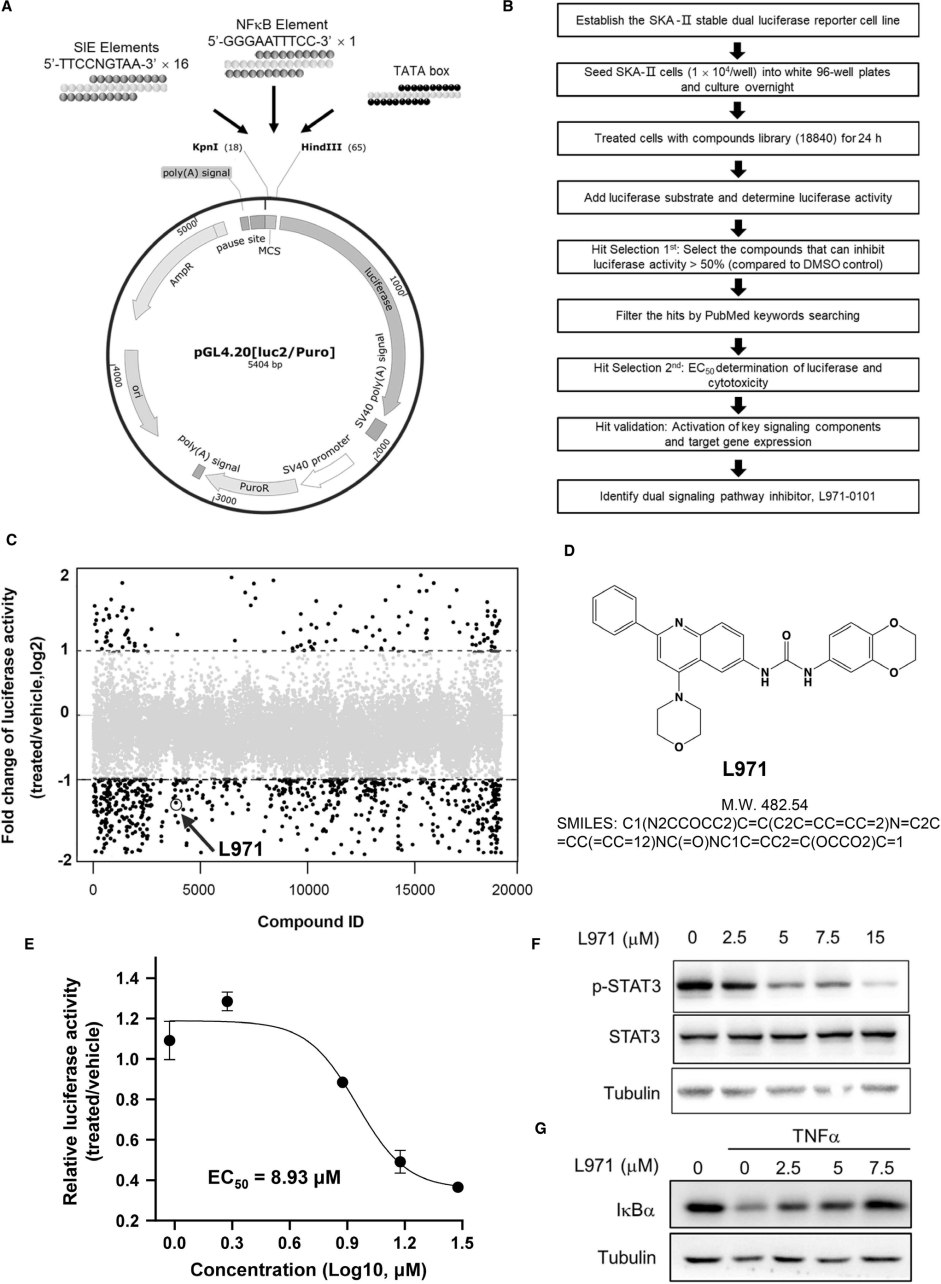
L971 was identified as an inhibitor of STAT and NFκB signalling pathways by a STAT and NFκB‐based luciferase drug screening system. A, Schematic view of the luciferase reporter plasmid, N represents C or T. B, Workflow of high‐throughput screening procedure. C, SKA‐II cells were seeded into 96‐well plates (1 × 10^4^/well) and cultured overnight. Cells were then treated with 18 840 individual compounds at 20 μM for 24 h, and luciferase activities were determined. The two black dotted lines represent the relative luciferase value of compound/vehicle treatment ranging from 0.5 to 2. D, The chemical structure, molecular weight and SMILES of L971. E, SKA‐II cells were seeded as in (C) and treated with vehicle or L971 at 0.9375, 1.875, 7.5, 15 and 30 μM. Luciferase activities after 24 h were determined after 24 h. F, A549 cells were treated with vehicle or L971 at 2.5, 5, 7.5 and 15 μM for 2 h. Whole cell lysates were processed for Western blot analysis and probed with anti‐pTyr705‐STAT3 and anti‐STAT3 antibodies. Tubulin was used as a loading control. G, THP‐1 cells were treated for 2 h with vehicle or L971 as in (F), followed by TNFα stimulation (20 ng/ml, 10 min). Whole cell lysates were processed for Western blot analysis and probed with anti‐IκBα antibodies. Tubulin was used as a loading control

To identify compounds that can effectively inhibit JAK/STAT and NFκB signalling, we constructed a luciferase reporter plasmid which contains both STAT and NFκB‐binding elements (Figure 1A) and stably transfected into human lung cancer cell line A549 to establish the dual target reporter system SKA‐ΙΙ. Similarly to previously published method,^23^, ^24^, ^25^ we established a high‐throughput drug screen workflow (Figure 1B) and total of 18 840 small molecules were screened (Figure 1C). Among them, 924 hit compounds exhibited more than 50% inhibition activities compared with vehicle at 24 hours whereas BP‐1‐102^26^ and Brevilin A^25^ which are known to inhibit STAT3 activity, shows 76% and 84% inhibition activity in our reporter system. These compounds were further filtered by searching PubMed database,^27^ and compounds previously reported to directly or indirectly interact with JAK/STAT and NFκB signalling were omitted. After that, total of 256 compounds were selected for further EC_50_ determination of luciferase activity and cytotoxicity (data not shown).

L971, a novel scaffold compound from Targetmol mini scaffold library, was identified as one of the most potent inhibitors in the reporter system (Figure 1C). From the PhysChem predictions,^28^ L971 fulfils the parameters of the Lipinski rule of five: MW = 482.54 (<500), LogP = 4.21 (<5), hydrogen bond acceptors = 8 (<10) and hydrogen bond donors = 2 (<5) and shows drug‐like properties. It is a bisarylurea compound containing morpholine ring and quinoline ring (Figure 1D) and could efficiently inhibit luciferase activity (EC_50_ = 8.93 µM) in a dose‐dependent manner (Figure 1E). The inhibition of luciferase activity might come from the decreased transcriptional activity of STAT or NFκB individually or both. To determine the possibility of JAK/STAT signalling inhibition, we measured L971 effects on the constitutive STAT3 phosphorylation in lung cancer cell line A549 (Figure 1F) and cervical cancer cell line HeLa (Figure S1A), as well as IL6‐induced STAT3 phosphorylation in HeLa cells (Figure S1B), indicating L971 inhibited STAT3 activation regardless of different genetic backgrounds, so did the FDA approved JAK inhibitors such as Ruxolitinib,^29^ Baricitinib^30^ and Tofacitinib^31^ (Figure S1C,D). Moreover, L971 could potently inhibit the phosphorylation of upstream kinases JAK1 and JAK2, with less extent of TYK2 in HeLa cells (Figure S1B). Meanwhile, L971 could also inhibit IκB degradation induced by TNFα (Figure 1G) and IKK phosphorylation induced by LPS (Figure S1E) in human monocytic leukaemia THP‐1 cells, indicating its inhibitory activity for NFκB signalling. These data suggest that L971 could function as a dual inhibitor of JAK/STAT and NFκB signalling pathways.

### 3.2 **L971 inhibits JAK/STAT and NF**κ**B signalling and downstream gene expression in peritoneal macrophage**

**FIGURE 2:**
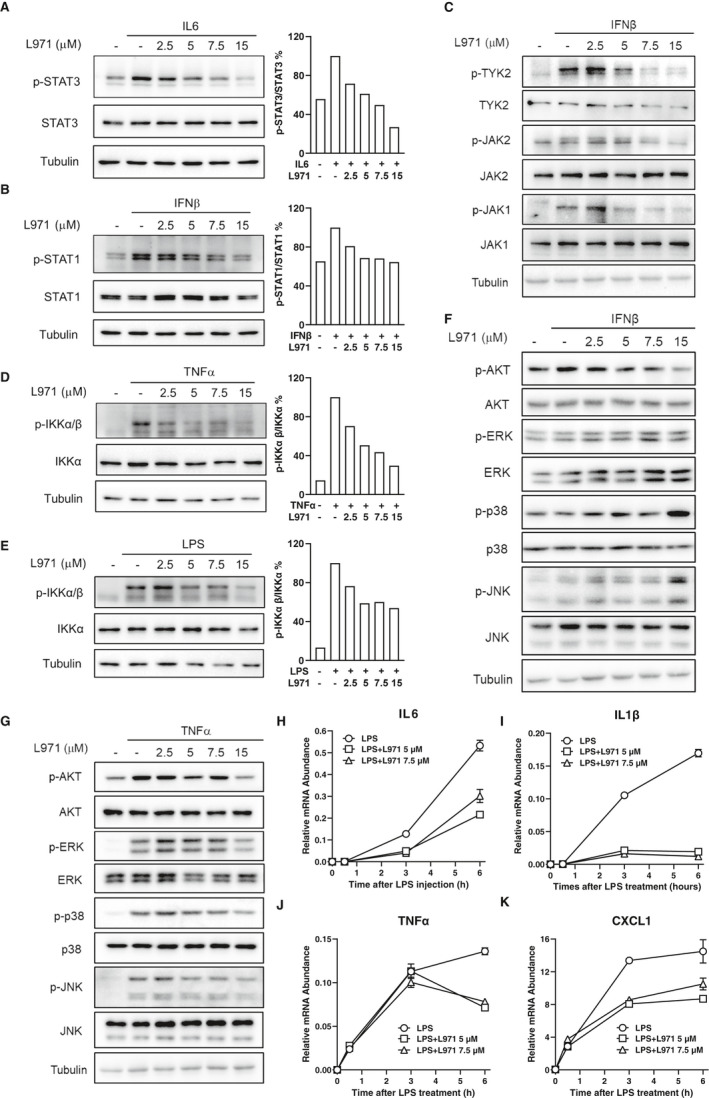
L971 inhibits JAK/STAT and NFκB signalling pathways and downstream gene expression in peritoneal macrophages. Peritoneal macrophages were isolated and treated with vehicle or L971 at 2.5, 5, 7.5 and 15 μM for 2 h, followed by stimulation with 20 ng/ml IL6 (A), 50 ng/ml IFNβ (B, C), 20 ng/ml TNFα (D) for 10 min or 100 ng/ml LPS (E) for 0.5 h. Whole cell lysates were processed for Western blot analysis and probed with primary antibodies including anti‐pTyr705‐STAT3 and anti‐STAT3 for (A), anti‐pTyr701‐STAT1 and anti‐STAT1 for (B), anti‐pTyr1054/1055‐TYK2, anti‐TYK2, anti‐pTyr1007/1008‐JAK2, anti‐JAK2, anti‐pTyr1022/1023‐JAK1 and anti‐JAK1 for (C), anti‐pSer176/180‐IKKα/β and anti‐IKKα for (D, E). Phosphorylation levels were normalized by corresponding total proteins and compared with vehicle group (right part of A, B, D, E). (F, G) Peritoneal macrophages were treated as in (B) for (F) and as in (D) for (G). Whole cell lysates were processed for Western blot analysis and probed with indicated primary antibodies including anti‐pThr308‐AKT, anti‐AKT, anti‐pThr202/Tyr204‐ERK, anti‐ERK, anti‐pThr180/Tyr182‐p38, anti‐p38, anti‐pThr183/Tyr185‐JNK and anti‐JNK antibodies. Tubulin was used as a loading control. (H‐K) Peritoneal macrophages (6 × 10^6^/60 mm dish) were pretreated with vehicle or L971 (5 μM or 7.5 μM) for 30 min and then stimulated with 100 ng/ml LPS for additional 0, 0.5, 3 and 6 h. The mRNA levels of IL6 (H), IL1β (I), TNFα (J) and CXCL1 (K) were determined by RT‐PCR

As JAK/STAT and NFκB signalling pathways serve as regulatory hubs that coordinate immune and inflammatory responses,^11^ we further detected the effects of L971 in general immune cells, such as peritoneal macrophages. Macrophage engagement in inflammatory responses could be directed and enhanced by cytokine‐induced JAK/STAT signalling (IL6 and type I interferon including IFNα and β)^4^, ^5^ and NFκB signalling (LPS and TNFα)^32^, ^33^ As shown in Figure 2A, L971 could inhibit IL6‐induced STAT3 activation in mouse primary peritoneal macrophages in a dose‐dependent manner. Similarly, L971 also dose‐dependently inhibited IFNβ induced STAT1 phosphorylation (Figure 2B) and the upstream kinase phosphorylation including JAK1, JAK2 and TYK2 (Figure 2C). Consistent with the inhibition of NFκB signalling in THP‐1 cells (Figure 1G and S1E ), L971 also inhibited IKK phosphorylation induced by TNFα (Figure 2D) and LPS (Figure 2E) in primary macrophage. To estimate whether other inflammation‐related signals were modulated by L971, we detect the phosphorylation of AKT, ERK, JNK and p38 upon IFNβ (Figure 2F) TNFα (Figure 2G) and LPS treatments (Figure S2). The phosphorylation of ERK, JNK and p38 was not inhibited by L971, whereas AKT activation was attenuated to some extent only in LPS challenged macrophages. As LPS could activate JAK/STAT and NFκB signalling sequentially,^34^ we measured their major downstream gene expression. L971 hindered the expression of IL6 (Figure 2H), IL1β (Figure 2I), TNFα (Figure 2J) and C‐X‐C Motif Chemokine Ligand 1 (CXCL1) (Figure 2K) induced by LPS challenge. These data indicated that L971 might exhibit anti‐inflammatory by suppressing JAK/STAT and NFκB pathways and their downstream gene expression in peritoneal macrophage.

### 3.3 Transcriptome profile of LPS‐induced proinflammatory response in peritoneal macrophage

**FIGURE 3:**
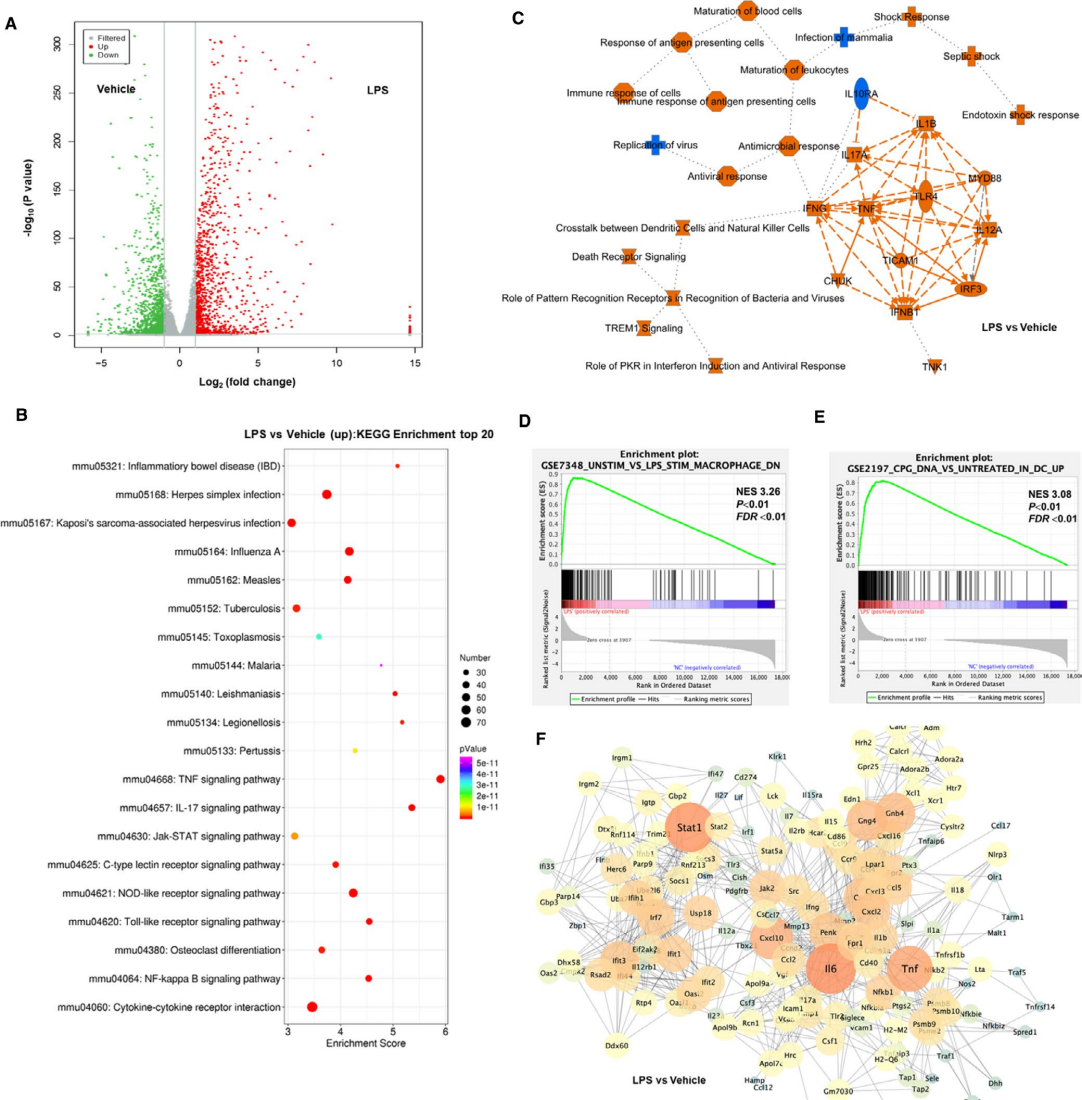
LPS induced proinflammatory response in peritoneal macrophages. A, The volcano plot of DEGs between LPS‐ vs vehicle‐treated groups. The grey dots represented genes without significant difference between two groups, the red dots showed those genes’ expression level was significantly up‐regulated and the green dots mean significantly down‐regulated in LPS‐treated group, compared with vehicle‐treated group. B, The top 20 category terms of KEGG analysis of up‐regulated DEGs between LPS and vehicle groups. C, The graphical summary of significant changes between LPS and vehicle group by IPA analysis. Symbols of target proteins, canonical signalling, immune cell regulation and diseases in red indicate a predicted increase or activation. The blue symbols indicate predicted decrease or down‐regulation. D, E, GSEA data sets enriched in LPS up‐regulated gene clusters. (F) PPI analysis of up‐regulated DEGs in LPS‐treated group

To better elucidate LPS induced inflammatory response, we performed RNA sequencing studies in LPS challenged peritoneal macrophage. There are 1389 significantly up‐regulated genes and 1365 significantly down‐regulated genes between control and LPS treatments (Figure 3A). Functional enrichment analysis by mapping DEGs to KEGG database indicates that LPS treatment could induce various proinflammatory signalling activities.^20^ Top 20 enriched pathways ranked by significance include TNF signalling pathway, JAK/STAT signalling pathway and NFκB signalling pathway, as well as inflammatory diseases, including inflammatory bowel disease (Figure 3B). Using IPA analysis,^35^ relationships between significant DEGs with disease and function were summarized (Figure 3C). Multiple inflammatory factors related to LPS stimulation were up‐regulated, including Toll‐like receptor 4 (TLR4), TNF, IL1β, IFNG (IFNγ), CHUK (IKKα) and interleukin 17A (IL17A) (in red colour), which matched well with RT‐PCR results shown in Figure 2E‐G. In agreement with Figure 3B, JAK/STAT and NFκB signalling‐related leukocyte activation and shock response were both enriched in this network (Figure 3C). Furthermore, GSEA was utilized to evaluate gene expression patterns at the level of published, classified gene sets in the Molecular Signatures Database.^36^, ^37^ And, the results clearly demonstrated that gene sets relationships such as GSE7348_UNSTIM_VS_LPS_STIM_MACROPHAGE_DN (NES 3.26, *P* < .05) and GSE2197_CPG_DNA_VS_UNTREATED_IN_DC_UP (NES 3.08, *P* < .05) were enriched in LPS up‐regulated genes (Figure 3D,E). In the PPI network,^38^ IL6, TNF, NFκB1, CXCL and STAT1 were identified as hub genes to regulate responses to LPS treatment (Figure 3F). In summary, transcriptome profiling results verify the main contribution of JAK/STAT and NFκB signalling in LPS‐induced proinflammatory responses in peritoneal macrophage.

### 3.4 Anti‐inflammatory effects of L971 were profiled by transcriptome sequencing

**FIGURE 4:**
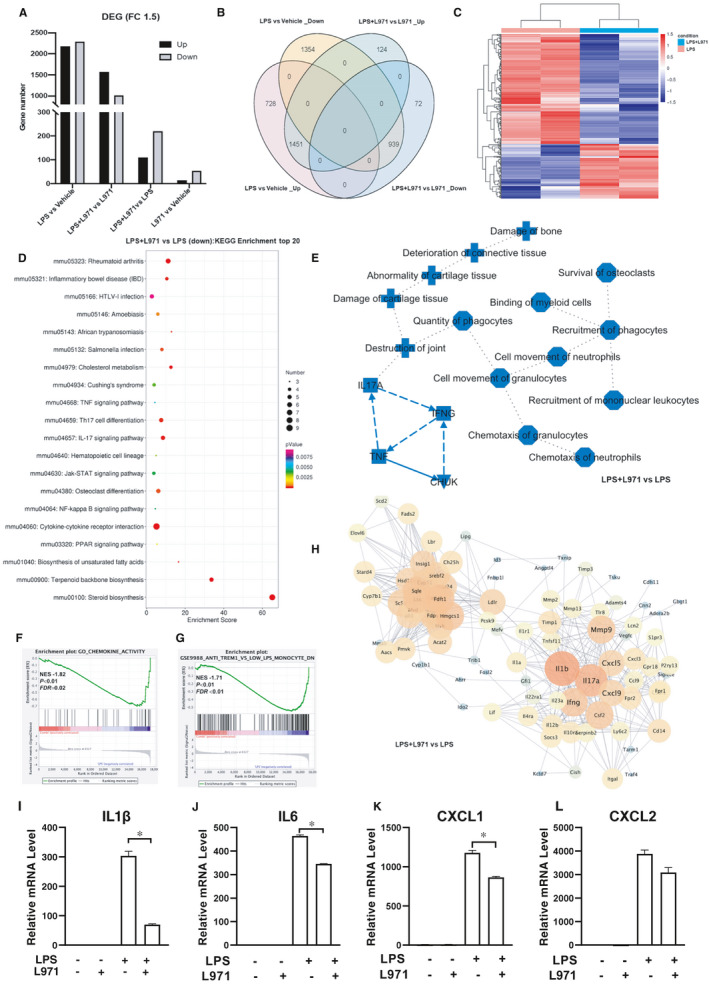
Anti‐inflammatory effects of L971. A, Bar chart of DEGs among different treatment groups. B, Venn diagram of DEGs among indicated comparison groups. C, Hierarchical cluster analysis of DEGs between LPS+L971 and LPS groups. The up‐regulated DEGs are indicated in red, and the down‐regulated DEGs are in blue. D, The top 20 category terms of KEGG analysis down‐regulated DEGs between LPS+L971 and LPS groups. E, The graphical summary of the significant difference between LPS+L971 and LPS groups by IPA analysis. Symbols of target proteins, canonical signalling, immune cell regulation and diseases in blue indicate a predicted decrease or down‐regulation. F, G, GSEA data sets enriched in LPS+L971 down‐regulated gene clusters compared with LPS alone. H, PPI analysis of LPS+L971 down‐regulated DEGs. FPKM levels of IL1β (I), IL6 (J), CXCL1 (K), CXCL2 (L) were graphed. Bars represent mean ±SEM, and *P < .05 is considered significant

To comprehensively investigate the anti‐inflammatory activities of L971, we performed transcriptome sequencing in peritoneal macrophages treated with LPS or LPS+L971. In L971 pretreated peritoneal macrophages, the number of LPS induced significant DEGs decreased in both up‐regulated DEGs and down‐regulated DEGs (Figure 4A, LPS vs Vehicle compare with LPS+L971 vs L971). The tendency of LPS induced up‐regulated DEGs was largely reversed, and only 1451 genes out of LPS induced 2179 up‐regulated DEGs keep significantly different in L971 pretreated comparison (Figure 4B, LPS+L971 vs L971_up compare with LPS vs Vehicle_up). Similarly, only 939 genes out of LPS induced 2293 down‐regulated DEGs maintain significantly different in L971 pretreated comparison (Figure 4B, LPS+L971 vs L971_down compare with LPS vs Vehicle_down). There are additional 124 up‐regulated DEGs (LPS+L971 vs L971_up compared with LPS vs Vehicle_up) and 72 down‐regulated DEGs (LPS+L971 vs L971_down compared with LPS vs Vehicle_down) exclusively enriched in the DEGs of L971 pretreated samples (Figure 4B). These data suggested L971 treatment might interfere LPS‐induced gene expression profiles. To assess the specific influence of L971 in LPS challenged macrophages, we further compared the gene expression differences between LPS‐treated peritoneal macrophages and LPS+L971‐treated counterparts. Enormous DEGs were identified and around two‐thirds of DEGs were significantly down‐regulated by L971 (Figure 4A,C, LPS+L971 vs LPS). In‐depth analysis of these down‐regulated DEGs revealed that TNF, JAK/STAT and NFκB signalling pathways were enriched in top 20 KEGG pathways (Figure 4D). Furthermore, cytokine‐cytokine receptor interaction (*P* < .05) was obviously down‐regulated after pretreatment with L971. The LPS induced inflammatory factors such as TNF, IFNG, CHUK (IKKα) and IL17A (Figure 3C), as well as myeloid cell function and inflammatory diseases, were predicted to be suppressed after L971 treatment in IPA analysis (Figure 4E). Consistently, the gene sets in GO_CHEMOKINE_ACTIVITY (NES −1.71, *P* < .05) and GSE9988_ANTI_TREM1_VS_LOW_LPS_MONOCYTE_DN (NES −1.82, *P* < .05) were enriched in L971 down‐regulated genes by GSEA analysis (Figure 4F,G). In the PPI network analysis of L971 down‐regulated DEGs, proinflammatory cytokines and chemokines such as IFNG, IL1β, IL17A, CXCL5, CXCL9 and matrix metallopeptidase 9 (MMP9), which are presented in the LPS up‐regulated hub genes (Figure 3F), were listed at the core of network (Figure 4H). Meanwhile, gene expression abundance of IL1β, IL6, CXCL1, CXCL2 of peritoneal macrophage significantly down‐regulated in response to L971 are shown in the histograms (Figure 4I‐L). All the above analysis indicates that L971 impairs LPS induced inflammation related signal transduction, mainly through JAK/STAT and NFκB signalling pathways.

### 3.5 Comparative illustration of L971 anti‐inflammatory efficiency by IPA

**FIGURE 5:**
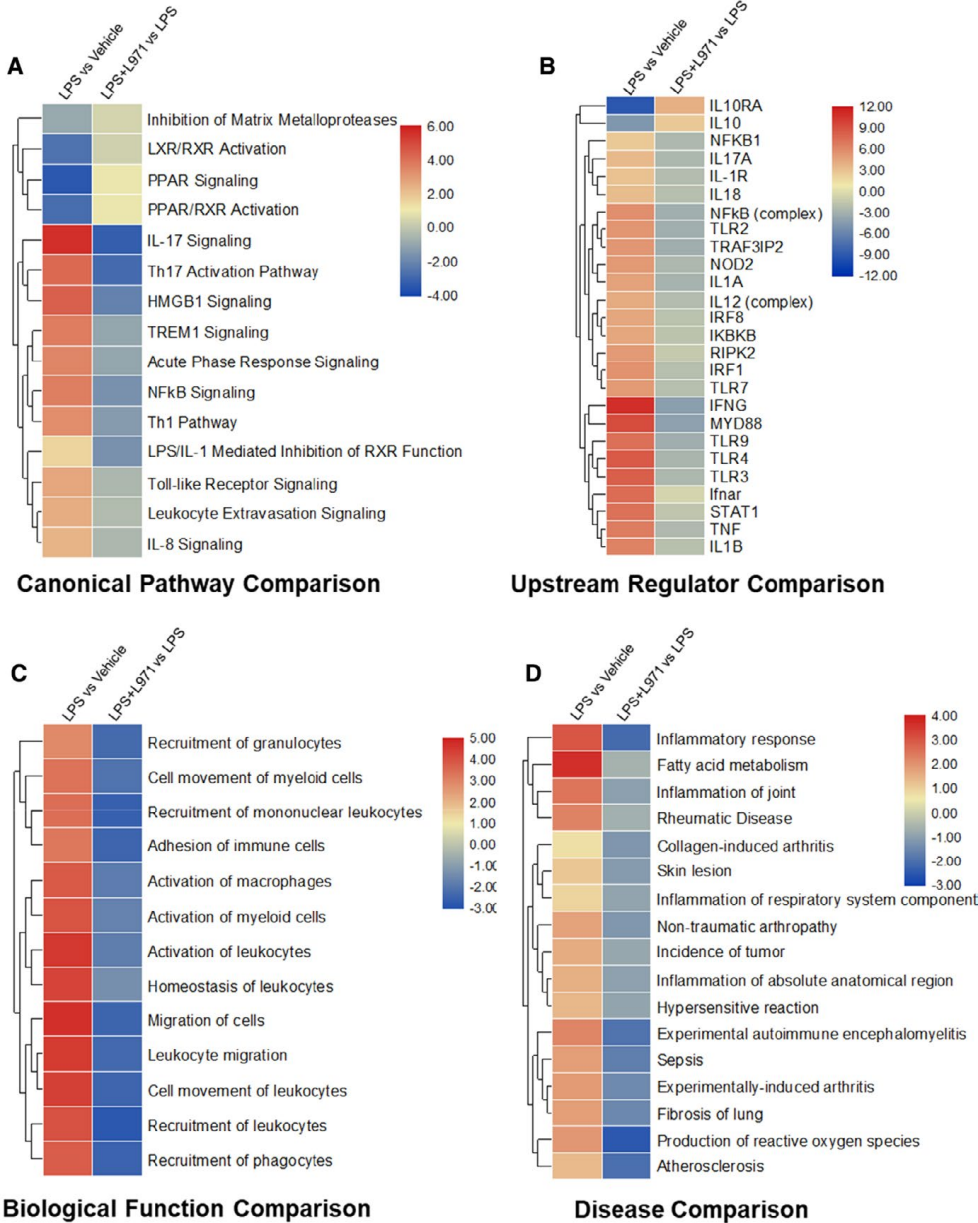
Comparative analysis of L971 anti‐inflammatory efficiency by IPA. (A) Canonical Pathway Comparison, (B) Upstream Regulator Comparison, (C) Biological Function Comparison and (D) Disease Comparison were conducted by comparing significant difference between LPS+L971 vs LPS and LPS vs vehicle. Positive z scores indicate an increase whereas negative ones indicate a reduction in specific terms

In order to further elaborate the influence of L971 from different aspects, we performed further analysis using IPA^35^ by comparative analysis. In canonical pathway comparison (Figure 5A), various signalling pathways up‐regulated by LPS were inhibited after treatment by L971, especially in proinflammatory signalling pathways, such as NFκB signalling, leukocyte extravasation signalling, acute phase response signalling, IL17 and interleukin 8 (IL8) signalling. In upstream regulator comparison, predicted upstream regulators, including NFκB1, IL17α, IL1R, NFκB (complex), toll like receptor 2 (TLR2), STAT1, TNF and IL1β, were down‐regulated by L971 (Figure 5B). Meanwhile, L971 up‐regulated critical anti‐inflammatory interleukin 10 (IL10) signalling limiting the immune response to pathogens to prevent excess host damage,^39^ which is down‐regulated in LPS treatment. In biological function comparison, L971 could inhibit the activation, migration and adhesion of immune cells including macrophages, myeloid cells, leukocytes and phagocytes in LPS‐induced inflammatory reactions (Figure 5C). Furthermore, in disease comparison, the majority of terms hindered by L971 treatment are related to inflammation diseases such as inflammatory response, rheumatic disease, inflammation of respiratory system component and sepsis (Figure 5D). All these data confirm that L971 could exert anti‐inflammatory effects through JAK/STAT and NFκB signalling pathways, indicating the possible applications in inflammation‐associated diseases like sepsis.

### 3.6 L971 treatment ameliorates endotoxin‐induced septic shock in vivo

**FIGURE 6:**
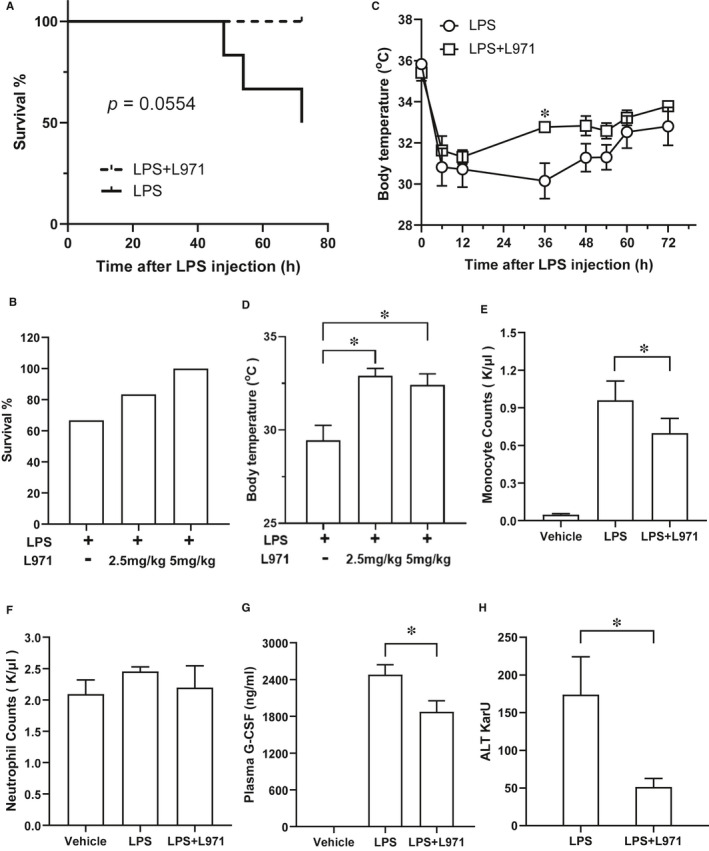
L971 ameliorates LPS induced septic shock in vivo. Mice were i.p. administrated with 5 mg/kg L971 12 h before LPS challenge (15 mg/kg, i.p.) and killed at 72 h after LPS injection. Survival rate (A) and body temperature (C) were recorded. In a separate experiment, mice were i.p. administrated with 2.5 mg/kg or 5 mg/kg L971 12 h before LPS challenge (15 mg/kg, i.p.) and killed at 36 h after LPS injection. Survival rate (B) and body temperature (D) were measured at 36 h after LPS treatment. Mice treated as in (A) were killed 72 h after LPS injection and monocyte (E) and neutrophil (F) counts in whole blood and plasma ALT levels (H) were measured. (G) Mice were i.p. administrated with 5 mg/kg L971 12 h before LPS challenge (15 mg/kg, i.p.) and killed at 6 h after LPS injection. The detection of plasma G‐CSF level was evaluated by ELISA assays

As a major cell wall component of gram‐negative bacteria, LPS is the major endotoxin inducing a systemic inflammatory response and plays a central role in sepsis.^40^ LPS‐induced sepsis shock is a well‐established animal model to mimic human sepsis pathology.^40^ Moreover, sepsis is one of the diseases predicted to be alleviated by L971 (Figure 5D). Based on the above assumption, we examined L971 protective efficiency in LPS induced mouse sepsis model in vivo. Upon a lethal dose of LPS injection (15 mg/kg), mouse survival was dramatically improved by intraperitoneal infusion of L971 (5 mg/kg) (Figure 6A) and survival rate rose accompanied with dose increasement (Figure 6B). L971 also had an obvious effect on the recovery of mouse body temperature, which is conducive to the mouse survival in sepsis shock,^41^ and amelioration especially dominates after 36 hours after LPS challenge (Figure 6C). Furthermore, the body temperature at 36 hours was monitored with various doses of L971 treatment. The results show that even lower dose of L971 (2.5 mg/kg) is sufficient to protect the mice from lethal lesion (Figure 6D). As shown in Figure 5C, activation of myeloid cells was predicted to be suppressed by L971. Therefore, we measured the counts of neutrophil and monocyte in mouse whole blood, and these two myeloid populations decreased in L971 treated group as predicted (Figure 6E,F). As G‐CSF is crucial for neutrophil activation contributing to tissue damage and organ dysfunction during early sepsis,^42^, ^43^ we measured plasma G‐CSF levels and data suggested that L971 could decrease the LPS induced G‐CSF levels (Figure 6G). Liver failure complication is recognized as one of the components that contribute to the severity of the sepsis shock.^44^ Plasma alanine aminotransferase (ALT) level is positively correlated with liver cell injury and widely used in the measurement of liver damage.^45^ ALT levels were potently reduced in L971 treated mice (Figure 6H). Collectively, these findings suggested that L971 had protective effects in LPS‐induced sepsis shock in vivo.

## 4 DISCUSSION

In recent years, the molecular mechanisms of inflammatory responses have been deeply elucidated.^46^ The JAK/STAT and NFκB signalling pathways are considered to play important roles in the transmission of inflammatory signals.^4^, ^5^ Multiple drugs targeting these two pathways were developed, and some of them have already been approved for the treatment of immune diseases such as inflammatory bowel disease, rheumatoid arthritis and non‐specific inflammation.^6^ As the frequent co‐occurrence of these two pathways, drugs simultaneously targeting both pathways was predicted to be more efficient for the treatment of inflammatory diseases.^10^, ^11^ For this purpose, we constructed a dual luciferase reporter system examining both JAK/STAT and NFκB activities and identified a new scaffold compound L971 as a dual inhibitor for JAK/STAT and NFκB signalling pathways. L971 inhibits phosphorylation of STAT1, STAT3 and upstream kinases JAK1, JAK2 and TYK2, as well as IKK phosphorylation and IκBα degradation in various cell types. Whole transcriptomic profile elucidates the anti‐inflammatory characteristics of L971 via JAK/STAT and NFκB signalling inhibition, analysed by various bioinformatic methods including KEGG enrichment, IPA prediction, GSEA evaluation and PPI network. Finally, we verified L971 protective activities in LPS‐induced sepsis in vivo.

Beyond their important regulatory functions in immune response, STAT3 and NFκB family members are ubiquitously expressed in most types of cancer and involved in tumorigenesis, progressions and metastasis.^47^, ^48^ Moreover, sepsis is significantly associated with increased risk for many cancers including chronic myeloid leukaemia, myelodysplastic syndrome, acute myeloid leukaemia, cancers of the cervix, liver, lung, rectum and colon.^49^ To date, multiple NFκB inhibitors were evaluated in clinical to treat various cancer types including prostate, renal and colorectal cancers, melanoma and leukaemia whereas the JAK/STAT inhibitors were also tested for their anti‐cancer efficacy in ovarian, brain, prostate, pancreatic and neurologic cancers and multiple myeloma.^6^ Our data show L971 is a dual inhibitor of JAK/STAT and NFκB and could ameliorate experimental sepsis shock, suggesting its potential usage for both cancer therapy and anti‐inflammatory diseases. By retrieving the structurally similar compounds of L971 through the Scifinder database,^50^ 22 analogues were identified (Score above 80 points). Among them, compound I4 discovered by Qingqing Guo et al have an extremely similar structure to L971 with proved anti‐tumour potency,^51^ suggesting the possible anti‐tumour activity of L971. The druggable potential of L971 and its analogue can be explored in future studies.

Besides the proved anti‐inflammatory and predicted anti‐tumour effects, L971 might participate in the cholesterol metabolism and steroid biosynthesis based on KEGG enrichment analysis (Figure 4D). PPI network (Figure 4H) also identifies a panel of hub genes related to fatty acid metabolism down‐regulated by L971 such as squalene monooxygenase (SQLE), farnesyl diphosphate farnesyl transferase (FDFT1), sterol regulatory element binding transcription factor 2 (SREBF2). Disease comparison employed by IPA further points out that fatty acid metabolism and atherosclerosis were improved by L971. Moreover, inflammation plays a key role in the initiation and development of metabolic diseases including obesity, type 2 diabetes, and atherosclerosis.^52^ Consistent with their central roles in coordinating inflammatory responses, numerous studies have implicated the activation of JAK/STAT and NFκB signalling in the pathogenesis of metabolic disease by both clinical and basic science research.^53^, ^54^ Taken together, L971 might contribute to regulation of metabolic disorders through direct influence of PPARγ signalling pathway (Figure 4D) or indirect interfering with JAK/STAT and NFκB signalling pathways.

For deeply dissect the molecular characteristics of L971, we employed online SwissTargetPrediction^55^ analysis searching for its possible targets within a larger collection of 376,342 compounds known to be experimentally active on an extended set of 3068 macromolecular targets.^56^ Total of 45 putative target proteins of L971 were retrieved, and target properties are widely distributed including protease, G protein‐coupled receptor, kinase, phosphatase and other proteins closely related to signal transduction (Figure S3A). To find out the possible connection of the predicted targets with the aforementioned JAK/STAT, NFκB and AKT signalling pathways, we build the direct communication network among them using IPA analysis (Figure S3B). There are 24 out of 45 predicted targets were directly related to JAK/STAT, NFκB and AKT signalling, including 5 G protein‐coupled receptors, 5 kinases, 3 proteases, 3 readers, 2 phosphatases and 6 other proteins, which provide a clue for further identification of L971 major targets.

In conclusion, we identified L971 as a novel anti‐inflammatory scaffold compound targeting both JAK/STAT and NFκB signalling pathways. This is in line with the current trend of developing multi‐targeting anti‐inflammatory drugs and has a great potential for further drug development.

## CONFLICT OF INTEREST

The authors have no conflicts of interest.

## AUTHOR CONTRIBUTION

**Mengyuan Li:** Formal analysis (equal); Investigation (supporting); Methodology (lead); Validation (lead); Writing‐original draft (equal). **Yu Yan:** Formal analysis (equal); Investigation (supporting); Methodology (equal); Validation (equal); Writing‐original draft (equal). **Xinxin Zhang:** Formal analysis (lead); Investigation (supporting); Validation (lead); Writing‐original draft (lead). **Yidan Zhang:** Writing‐review & editing (equal). **Xiaohan Xu:** Resources (equal). **Lei Zhang:** Methodology (supporting). **Liangliang Lu:** Methodology (supporting). **Jie Wang:** Methodology (supporting). **Yazhuo Zhang:** Methodology (supporting). **Qiaoling Song:** Conceptualization (equal); Data curation (lead); Investigation (equal); Visualization (lead); Writing‐review & editing (equal). **Chenyang Zhao:** Conceptualization (lead); Data curation (lead); Investigation (lead); Writing‐review & editing (lead).

## Supporting information

Fig S1

Fig S2

Fig S3

Table S1‐S2

Supplementary Material

## Data Availability

The data that support the findings of this study are available from the corresponding author upon reasonable request.
