# Supplementary figures and images for: Scaffold compound L971 exhibits anti‐inflammatory activities through inhibition of JAK/STAT and NFκB signalling pathways
Source: J Cell Mol Med. 2021 May 20;25(13):6333–47. doi: 10.1111/jcmm.16609 (PMC8256347; doi:10.1111/jcmm.16609)

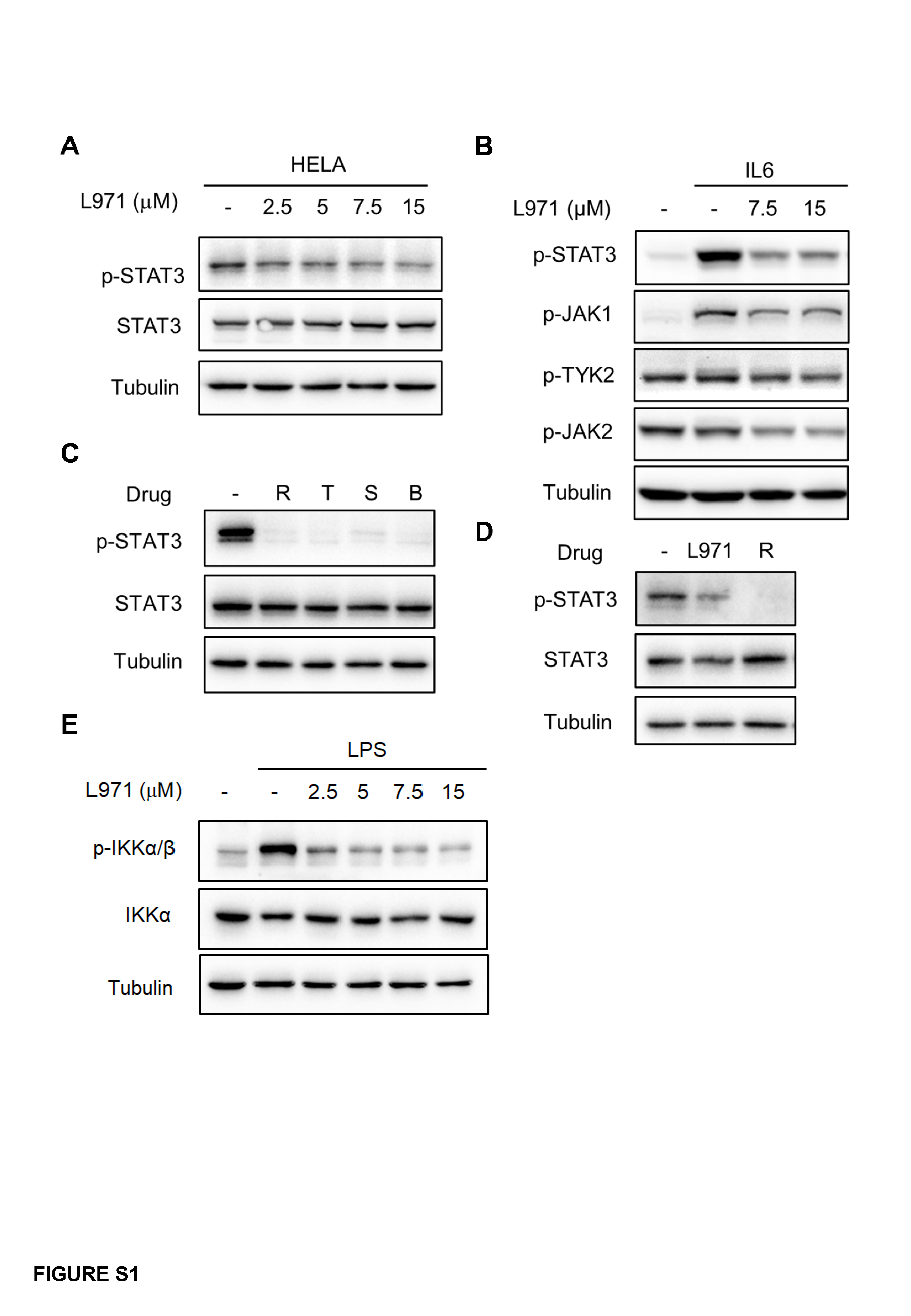

Supplement: Supplementary file 1 — Fig S1 [file JCMM-25-6333-s001.jpg]

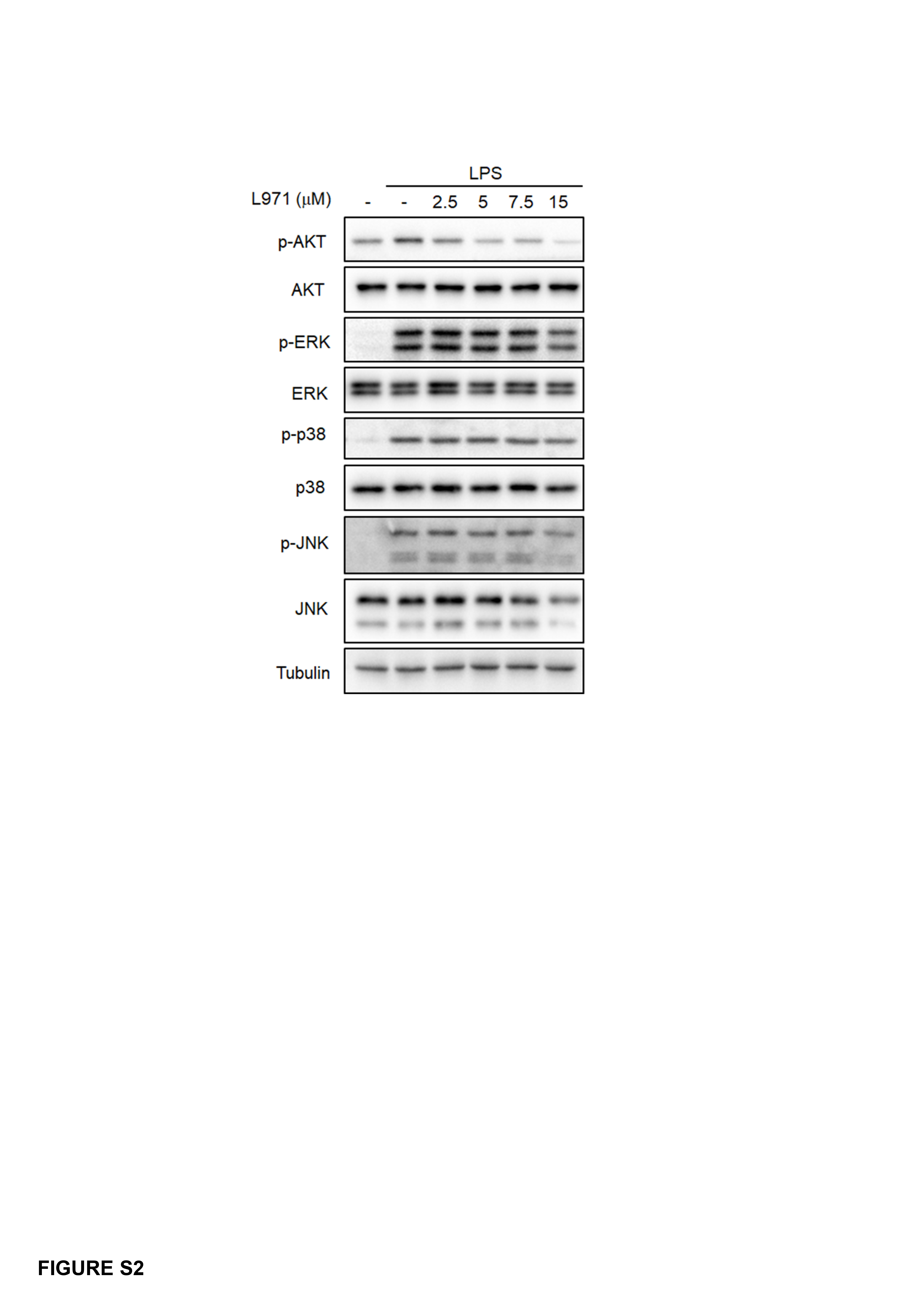

Supplement: Supplementary file 2 — Fig S2 [file JCMM-25-6333-s004.jpg]

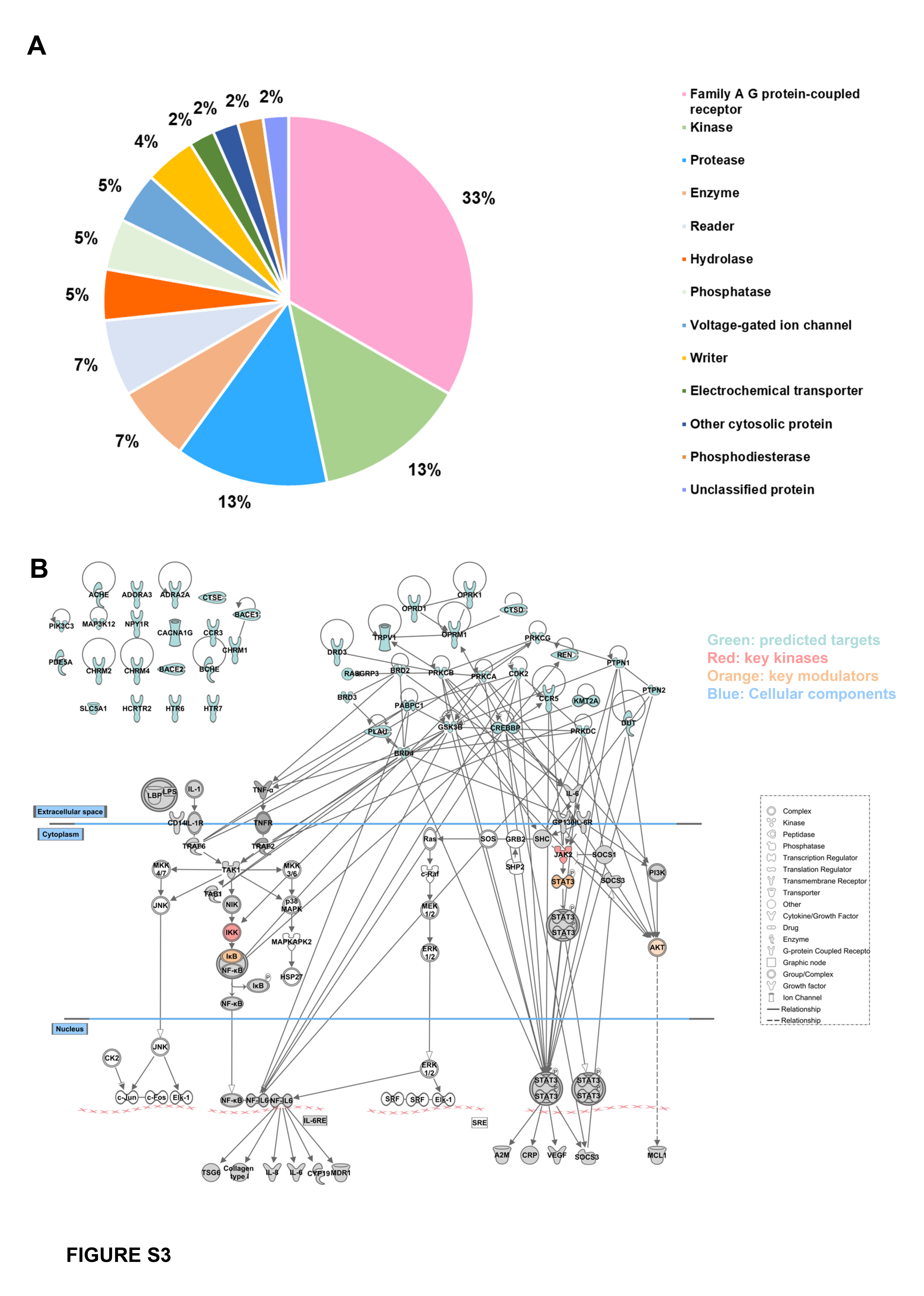

Supplement: Supplementary file 3 — Fig S3 [file JCMM-25-6333-s002.jpg]
