# Supplementary material for: Scaffold compound L971 exhibits anti‐inflammatory activities through inhibition of JAK/STAT and NFκB signalling pathways
Source: J Cell Mol Med. 2021 May 20;25(13):6333–47. doi: 10.1111/jcmm.16609 (PMC8256347; doi:10.1111/jcmm.16609)
Supplement: Supplementary file 4 — Table S1‐S2 [file JCMM-25-6333-s003.docx]

**Table S1：List of Antibodies**

| **Antibody name** | **Catalog No.** | **Company** | **Dilution** |
| --- | --- | --- | --- |
| Anti-pTyr701-STAT1 | 9167 | Cell Signaling Technology | 1:1000 |
| Anti-STAT1 | 14994 | Cell Signaling Technology | 1:1000 |
| Anti-pTyr705-STAT3 | 9145 | Cell Signaling Technology | 1:1000 |
| Anti-STAT3 | 9132 | Cell Signaling Technology | 1:1000 |
| Anti-pSer176/180-IKKα/β | 9740 | Cell Signaling Technology | 1:1000 |
| Anti-IKKα | 2682 | Cell Signaling Technology | 1:1000 |
| Anti-pTyr1022/1023-JAK1 | 3331 | Cell Signaling Technology | 1:1000 |
| Anti-JAK1 | 3332S | Cell Signaling Technology | 1:1000 |
| Anti-pTyr1054/1055-TYK2 | 9321 | Cell Signaling Technology | 1:1000 |
| Anti-TYK2 | 9312 | Cell Signaling Technology | 1:1000 |
| Anti-pTyr1007/1008-JAK2 | 3776 | Cell Signaling Technology | 1:1000 |
| Anti-pThr308-AKT | 9275 | Cell Signaling Technology | 1:1000 |
| Anti-AKT | 4691 | Cell Signaling Technology | 1:1000 |
| Anti-pThr202/Tyr204-ERK | 4370 | Cell Signaling Technology | 1:1000 |
| Anti-ERK | 4695 | Cell Signaling Technology | 1:1000 |
| Anti-pThr180/Tyr182-p38 | 4511 | Cell Signaling Technology | 1:1000 |
| Anti-p38 | 8690 | Cell Signaling Technology | 1:1000 |
| Anti-pThr183/Tyr185-JNK | 4668 | Cell Signaling Technology | 1:1000 |
| Anti-JNK | 9252 | Cell Signaling Technology | 1:1000 |
| Anti-IκBα | 4814 | Cell Signaling Technology | 1:1000 |
| Anti-Tubulin | sc-5286 | Santa Cruz | 1:5000 |

**Table S2：Description of Compound Libraries**

| **Drug library ID** | **Number of included compounds** | | **Resource company** | | **Catalog No.** | **Web links** | |
| --- | --- | --- | --- | --- | --- | --- | --- |
| Bioactive Compounds Library Plus | | 3187 | TargetMol | D7800 | | https://www.tsbiochem.com/library/  Bioactive%20Compounds%20Library%20Plus | |
| Mini Scaffold Library | | 4131 | TargetMol | L5600 | | https://www.tsbiochem.com/library/  Mini-Seleton-Library | |
| Approved Drug Library | | 1813 | TargetMol | L1000 | | https://www.tsbiochem.com/library/  Approved-Drugs-Library | |
| Clinical Compound Library | | 514 | TargetMol | L3400 | | https://www.tsbiochem.com/library/  Clinical-Compound-Library | |
| Bioactive Compound Library | | 830 | TargetMol | L4000 | | https://www.tsbiochem.com/library/  Bioactive-Compounds-Library | |
| Natural Compound Library | | 439 | TargetMol | L6000 | | https://www.tsbiochem.com/library/  Natural%20Compound%20Library%20for%20HTS | |
| BioBioPha Compound Library | | 3520 | The National Center for Drug Screening | NA | | The detailed compound information is available upon request |  |
| Analyticon Discovery | | 4406 | The National Center for Drug Screening | NA | | The detailed compound information is available upon request |  |
