# Supplementary material for: Scaffold compound L971 exhibits anti‐inflammatory activities through inhibition of JAK/STAT and NFκB signalling pathways
Source: J Cell Mol Med. 2021 May 20;25(13):6333–47. doi: 10.1111/jcmm.16609 (PMC8256347; doi:10.1111/jcmm.16609)
Supplement: Supplementary file 5 — Supplementary Material [file JCMM-25-6333-s005.docx]

**FIGURE S1. L971 inhibits JAK/STAT and NFκB signaling pathways.** **(A)** HeLa cells were treated with vehicle or L971 at 2.5, 5, 7.5 and 15 μM for 2 h. Whole cell lysates were processed for Western blot analysis and probed with anti-pTyr705-STAT3 and anti-STAT3 antibodies. **(B)** HeLa cells were treated for 2 h with vehicle or L971 at 7.5 and 15 μM, followed by IL6 stimulation (20 ng/ml, 10 min). Whole cell lysates were processed for Western blot analysis and probed with anti-pTyr705-STAT3, anti-pTyr1022/1023-JAK1, anti-pTyr1054/1055-TYK2, and anti-pTyr1007/1008-JAK2 antibodies. **(C)** DU145 cells were treated with Ruxolitinib (R), Tofacitinib (T), S-Ruxolitinib (S), and Baricitinib (B) at 10μM while HeLa cells were treated with L971 at 15 μM or R at 10 μM (**D**). Whole cell lysates were processed for Western blot analysis and probed with anti-pTyr705-STAT3 and anti-STAT3 antibodies. **(E)** THP-1 cells were treated for 2 h with vehicle or L971 at 2.5, 5, 7.5 and 15 μM, followed by LPS stimulation (100 ng/ml, 0.5 h). Whole cell lysates were processed for Western blot analysis and probed with anti-pSer176/180-IKKα/β and anti-IKKα antibodies. Tubulin was used as a loading control.

**FIGURE S2. L971 did inhibit phosphorylation of ERK, p38 and JNK but inhibited AKT in LPS treated peritoneal macrophages.** Peritoneal macrophages were isolated and treated with vehicle or L971 at 2.5, 5, 7.5 and 15 μM for 2 h, followed by stimulation with 100 ng/ml LPS for 0.5 h. Whole cell lysates were processed for Western blot analysis and probed with indicated primary antibodies including anti-pThr308-AKT, anti-AKT, anti-pThr202/Tyr204-ERK, anti-ERK, anti-pThr180/Tyr182-p38, anti-p38, anti-pThr183/Tyr185-JNK, anti-JNK antibodies. Tubulin was used as a loading control.

**FIGURE S3. Evaluation of predicted targets of L971 with JAK/STAT and NFκB signaling.** (**A**) The pie chart shows the class distribution of the putative target proteins of L971 based on SwissTargetPrediction analysis. (**B**) The direct connection of 45 predicted targets with JAK/STAT and NFκB signaling using IPA analysis.
